# A collaborative escape room as gamification strategy to increase learning motivation and develop curricular skills of occupational therapy students

**DOI:** 10.1186/s12909-021-02973-5

**Published:** 2021-10-27

**Authors:** Julia Dugnol-Menéndez, Estíbaliz Jiménez-Arberas, María Luisa Ruiz-Fernández, David Fernández-Valera, Allen Mok, Jesús Merayo-Lloves

**Affiliations:** 1 Padre Ossó Faculty, University of Oviedo, Prado Picón, S/N, 33008 Oviedo, Asturias Spain; 2 Faculty of Medicine and Health Sciences, University of Oviedo, Julián Clavería, 6, 33006 Oviedo, Asturias Spain; 3 California State Polytechnic University, Pomona, Pomona, USA

**Keywords:** Occupational therapy, Education, Escape room, Gamification, Students, Collaborative, Teamwork

## Abstract

**Background:**

This study presents an experiment regarding the introduction of gamification strategies in occupational therapy courses. Based on previous studies, the objective is to adapt the idea of recreational escape rooms to educational environments of health sciences like occupational therapy to increase student motivation and promote game-based learning and key skills, such as teamwork.

**Methods:**

Computer software was created for a collaborative escape room which allows on-line simultaneous play of up to 24–30 students. It was tested three times in an occupational therapy degree program with 75 students and it was based on two different subjects, although it can be adapted to others. The escape room was evaluated using feedback surveys and comparing students’ performances before and after the game. Descriptive exploratory statistical analysis was performed using SPSS version 24.

**Results:**

An appropriate use of educational escape rooms can have significant positive impacts on students’ engagement and learning. Students were found to prefer using gamification tools in their learning. Their degrees of satisfaction exceeded their expectations.

**Conclusions:**

Educational escape rooms may have a positive impact on students’ motivation and a statistically significant improvement of test scores after playing was found. Comments from the feedback surveys were used to improve successive versions of the software and design of the game.

**Trial registration:**

T.F.G. n° 2020.038 (Research Ethics Committee of the Principality of Asturias).

## Introduction

In recent years, many proposals have been made to improve students’ motivation [1]. One of the tools that has most interested the teaching community, as well as the health sciences, is gamification [2]. The term gamification refers to the use of elements of games in non-recreational situations, such as in a classroom [3]. These types of techniques are being introduced in the educational field, giving rise to what is known as educational gamification [4].

An important role of higher education is to foster the development of individuals’ social and curricular skills, goals that are difficult to reach with traditional methods [5]. Due to the heavy focus on short-term goals which, once achieved, are soon forgotten, it is the teacher’s responsibility to help students put into practice their basic knowledge of anatomy while studying related subjects and develop the necessary skills for their professional development [6]; therefore, students could benefit from gaming in their education and experiential learning for not only gaining knowledge but skills and attitudes as well [7].

Gamified activities aim to influence student behaviour while increasing concurrent enjoyment of learning, and consequently, the academic performance and motivation of the students [8]. When teaching anatomy laboratory sessions, learning becomes more effective if students work as a team, express opinions and thoughts and work together when problem solving [9]. A type of educational playful team activity that meets the aforementioned requirements, helps students in their learning and allows the development of their curricular skills, are escape rooms [10]. Escape rooms are described by Nicholson [11] as “live-action team-based games where players discover clues, solve puzzles, and accomplish tasks in one or more rooms, in order to accomplish a specific goal, usually escaping from the room, in a limited amount of time” (p.1). This offers a non-traditional, experiential, peer-group learning opportunity that fosters constructive interactions, leading to observations of one’s own and others’ unique leadership skills and styles [10]. Educational escape rooms put students in direct contact with each other and require them to collaborate; therefore, they are excellent activities to enhance an in-person classroom setting and, due to the characteristics of the escape game, cooperative and collaborative skills are enhanced during play [12].

An educational escape room proposal should be based on an original design, with clear objectives: to promote the enjoyment of learning and consequently the academic performance, to improve the motivation of the students to whom it is addressed and to develop important skills related to leadership capacity, teamwork, critical capacity, and communication [11]. Communication can be said to be an important skill in all areas of health science practice, and it is the most emphasized skill in literature [13]. Moreover, clinical environments and escape rooms share similarities. Learning becomes an enjoyable experience and leads to engaging health science students [14]. These types of games are ideal as a learning tool or to complement the skill set of members of clinical teams. The games provide a supportive safe and dynamically engaging environment, necessary for the development of occupational therapists [15–17].

In general, the use of the escape room as an experience of educational gamification has high utility and it helps to foster the enjoyment of learning, and consequently, the academic performance and motivation of the students [18–20]. These are some of the reasons why literature regarding educational escape rooms in higher education is increasing in different fields [21, 22]. However, nothing from occupational therapy is found.

### Study aims

The aims of this study were firstly to generate a situation in which the transversal contents of two different subjects (“Anatomy” and “Autonomy and functional independence in the adult”) within the occupational therapy degree program were integrated into an escape room activity to increase student motivation and knowledge of these subjects while also promoting the use of curricular skills and attitudes. Among other things, the choice of subjects is due to the relevance that students and professionals have always given to knowledge of anatomy for their job performance [6, 23]. The practical application of the collaborative escape room to the improvement of individual and group learning will be used as an indicator.

Secondly, it was necessary to find a method to evaluate the impact of this activity in terms of the students’ perceptions of its effectiveness and design.

## Material and methods

### Ethics statement

Students’ participation was voluntary, and the activity was performed with the informed consent of the participants. They were informed about the nature of the study in which a new instructional method was being researched for the benefit of the students. Data analysis was anonymous. The ethics committee at our institution (Research Ethics Committee of the Principality of Asturias) reviewed and approved the project due to the educational nature of the study (T.F.G. n° 2020.038).

### Sample selection

Therapystein© escape room was played three times with 25 different occupational therapy students each time. The summary of the whole process is shown in Table 1. The first time, the prototype was used. This was done with second-year occupational therapy students in the subject of Autonomy and functional independence in the adult. There was full participation (*n* = 25, 100%). The second time, the escape room was tested with first-year Anatomy students. There was 50% participation of students (n = 25, 50%). The third time, the escape room was played during the following academic year, this was done with second year students, with 100% of the students participating (n = 25, 100%). The reason that all the first-year students did not participate was due to a change in the date, time, and place of the game, which made it difficult for them to attend.

**Table 1:** Summary of the process

**Step 1: Design of the escape room**
• Brainstorming that includes: selection of content and tests, creation of materials and story, space study and room selection.
• Design of the software and App.
• Survey’s design.
• Select the timetable for playing.
**Step 2: Room design**
• Riddle recording.
• Storyboard and ridddle connection.
• Game master script.
• Scenario design and room setting.
• Commissioning of computer equipment and network connections.
**Step 3: Game day**
• Informed consent and rules for the students.
• Pre - test.
• Final settings of the scenario.
• Play the escape room.
• Post - test (individual and in groups).
• Feedback survey.
**Step 4: Evaluation and data analysis**

### Design and development of test

A test was developed to check the level of right answers before and after the game in understanding and correctly defining concepts related to the theoretical-practical content of the subjects. The tests were carried out twice, first individually and then in groups. This allowed us to observe and measure when students shared knowledge working as a team. It also promoted development of skills and values of group behaviour that are essential for professionals in the health sciences [24, 25].

### Design of the escape room

The escape room for occupational therapy students was developed to be implemented at the facilities of the university, but it could be adapted to any installation. The escape room was designed to test technical skills and anatomical and clinical knowledge, but also hidden objects, puzzles and riddles were used to emulate a traditional escape room.

**Fig. 1:**
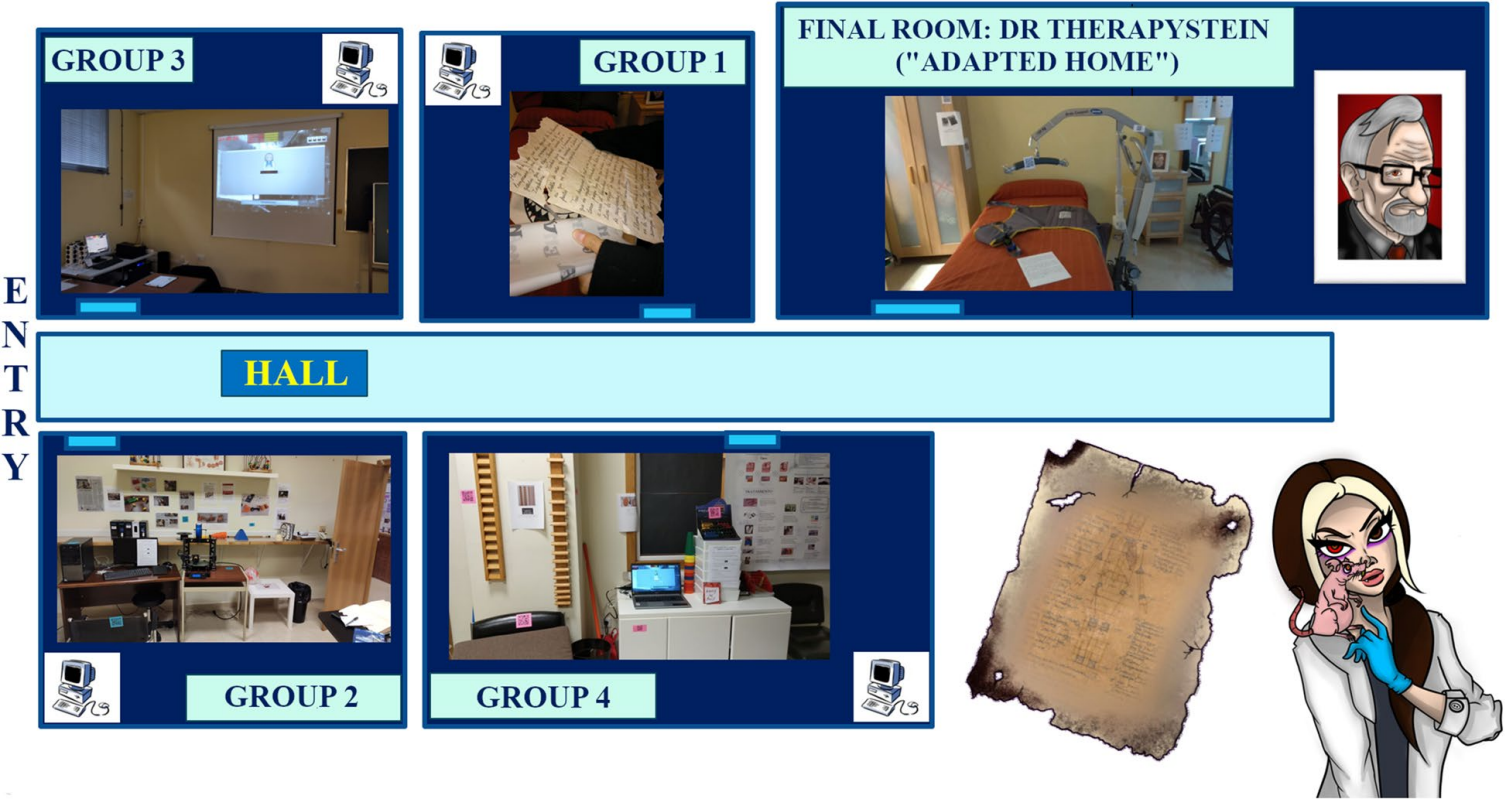
Main play area diagram. Step 1: Each group is inside its room solving clues and chatting with other groups. Step 2: Groups can go to the hall to continue the game, after unlocking all the program locks. Step 3: Students go to the final room to find the last clue to stop the timer of each room. Step 4: Students enter the last password to finish the game

To participate in the game, students were organised randomly into four groups of six players using a puzzle determined in which room they were assigned to. Groups were distributed between rooms, with one organizer in each to observe the participants’ progress throughout the challenge and to record the team collaboration strategies used. The final room, which was a home simulation laboratory (hereinafter called “adapted home”) was empty at the beginning of the game (see Fig. 1).

In each room, students could see a computer running the program (Therapystein®© was made with Unity (version 2018.3.5F1 personal) and C# (Microsoft Visual Studio 2017 Community, version 15.9.6)). When students pushed the “play” button, an introductory video started and explained the scenario, introducing the main characters (character designs were made with a graphic drawing tablet (XP-Pen Artist 15.6) using Gimp 2.10.8 and an iPad 2018 Apple Pencil, first generation, with the Procreate application; video was made using Sonie Movie Studio 13.0, 2014). The computers needed to be a part of a network, either through the web or locally.

Therapystein requires students to escape from the machinations of an evil villain, Dr. Therapystein. In order to escape, students must work together to solve problems using previously-learned knowledge. The students not only have to interact with members of the same team, but also have to interact with opposing teams. As the game progresses, the students are subsequently more and more immersed in the twisted world of Dr. Therapystein.

**Fig. 2:**
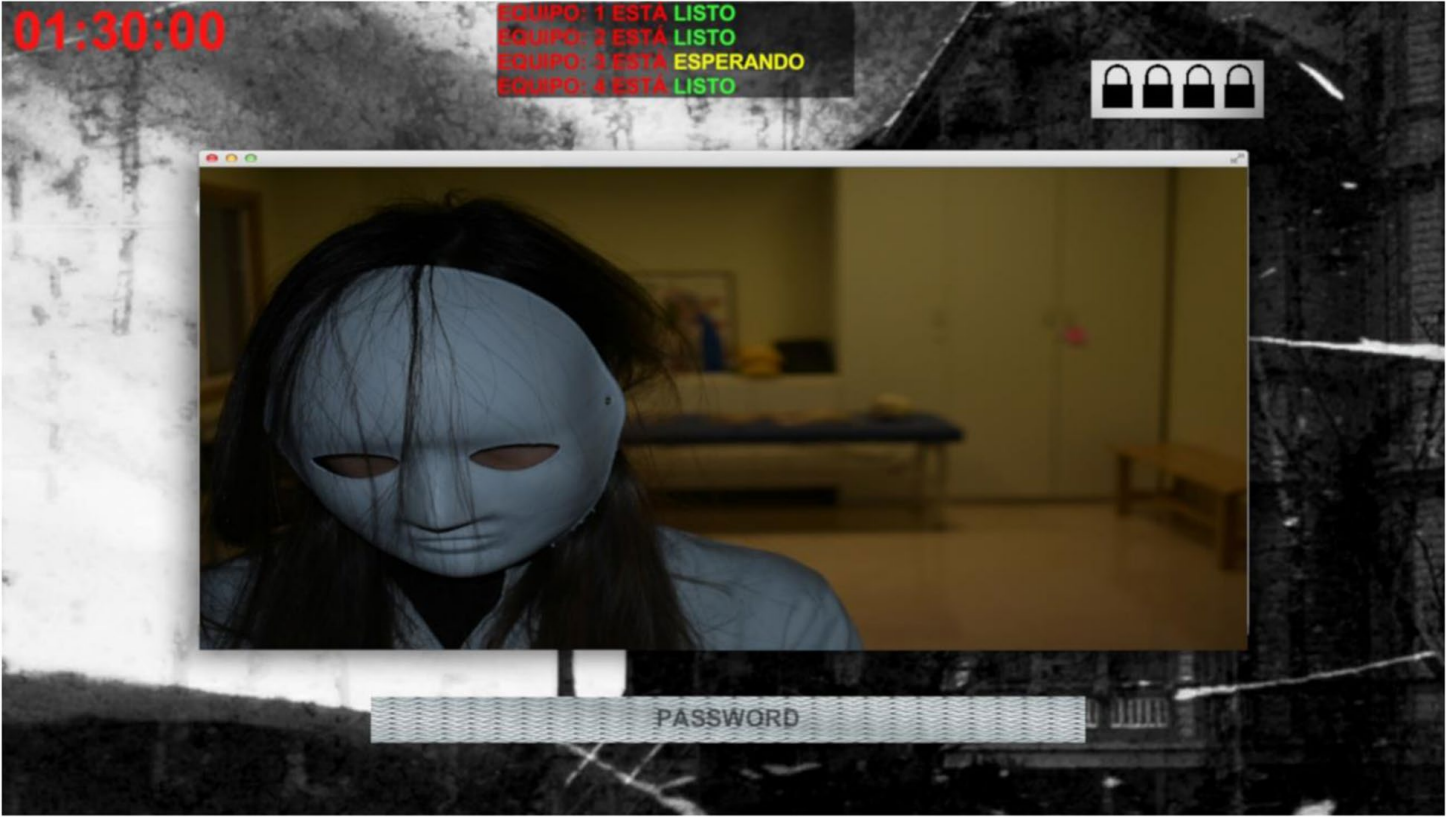
Screenshot of the game. Taken after watching the introductory video and waiting for team 3 to finish watching the video to start the game (Spanish status box)

When the video finished, the game and a countdown timer began simultaneously in all four rooms. Following this, an informative image and a box for entering a password appear on the screen (see Fig. 2). At the top right of the image four locked padlocks are located that indicate inaccessibility of the four rooms where students are located (for safety reasons, students were not actually locked in their rooms).

**Fig. 3:**
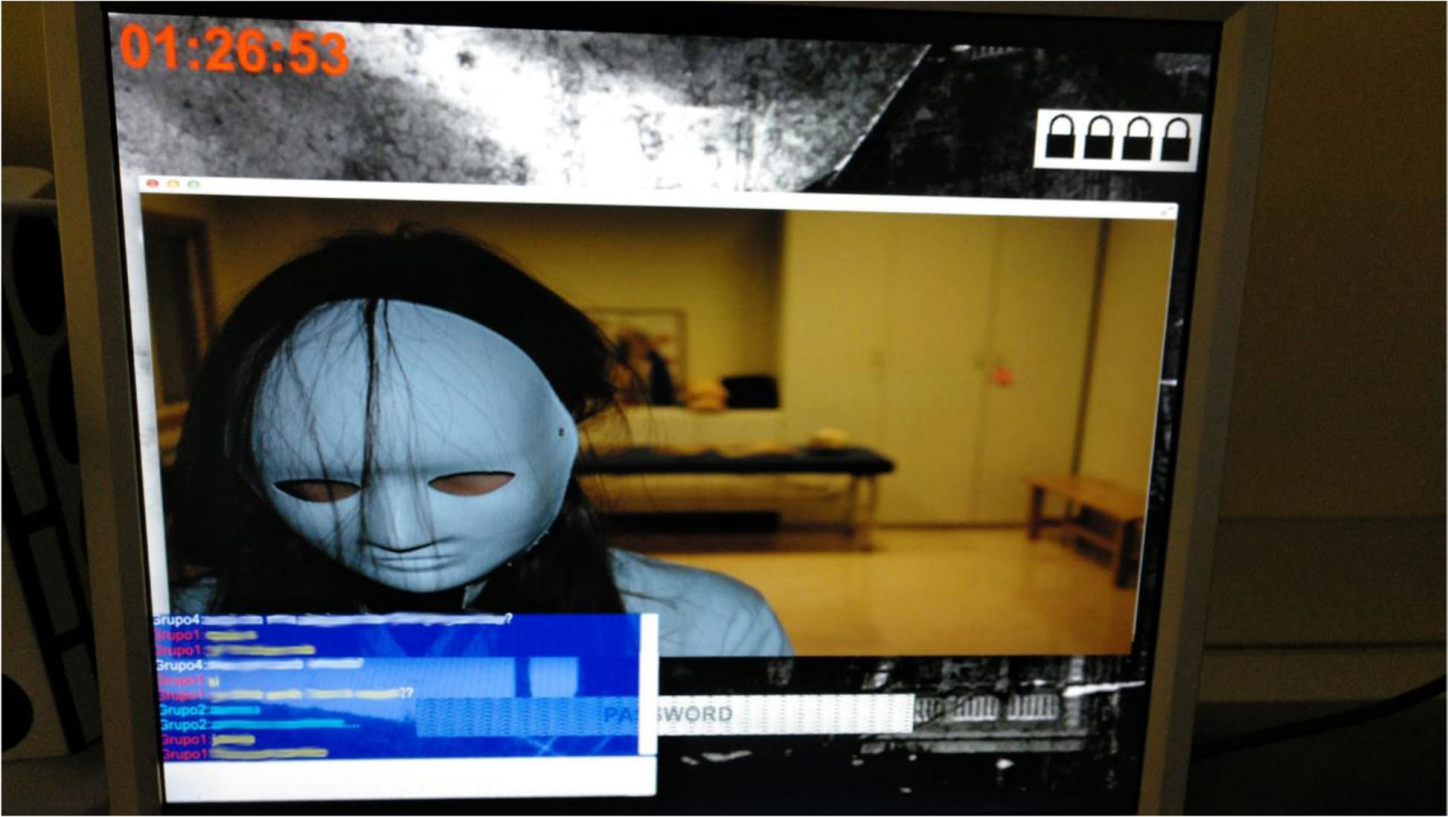
Chatbox to promote communication and collaboration between groups

One of the improvements compared to the game prototype was that the students in one room could communicate with other rooms through the in-game chat in such a way that the game went from being competitive to collaborative in its entirety. In this way they could pool their knowledge and solve problems together, to reach a common goal (see Fig. 3).

The first part of the game was sequential and partially guided in order to make it easier since most students were only novices at playing escape rooms. The first clues given were instructions found inside an envelope, and they were not allowed to use mobile phones at first. In addition to the instructions, the envelope contained some wild cards that could be delivered to the organizer in exchange for a hint or clue, although that resulted in penalty for usage in the final score. This was an important design feature as it helped ensure that teams could progress through the game and participate in all aspects of the educational activity.

Near the envelope was another clue: a sheet of newspaper with an anatomical and clinical term crossword puzzle. The students had to solve the crossword to obtain a password that had to be entered into the program. Following this, a clinical case appeared on the screen, and they had to answer the questions posed and give them to the “doctor’s assistants” for further instructions. They then needed to find an object related to the occupational therapy rehabilitation of that patient in the room. Successfully finding it allowed students to use their phones and a free application of augmented reality (HP AR Reveal app) to find new clues and riddles. From here, the game was no longer linear, and they could explore everything for new clues. There are two differences between the escape game prototype and the final version. Firstly, there was the use of a QR scanner (Therapystein visor©) in the final version instead of the HP AR Reveal app, that was used in the prototype. Secondly, in the final version, the clinical case was fragmented and hidden within QR clues, because it allowed more students to participate and collaborate.

To remove the computer program lock and continue the escape in the adapted house, they had to find a box with a four-digit padlock. The code to open it was hidden in the form of a riddle in an augmented reality or QR clip; they had to answer a series of anatomical questions correctly to get the combination of the padlock.

Inside the box, there was a note with an explanation, a clue with some letters of a sentence written on tracing paper and a password for unlocking the computer program. Students could see in real time if their classmates were able to open the other rooms since the program showed when the locks were opened. When every padlock of the program was unlocked, two students from each room could go out to the hall while their classmates continued in their room. It was decided to change this part of the game to make it more dynamic so, in the modified version, all students could go outside to the hall and dressing room to find clues to escape. But at the end, in all escape rooms, they needed to collaborate and sum up their clues to find the key that opened the final room. Once they found the key and entered the house, they saw a note from Mr. Therapystein’s diary about a day of his medical consultations. It named the characters of the students in each clinical case, alluding to objects found in the adapted house. A chest was hidden in the kitchen with a letter-lock. The code for that lock was in the utensils and objects that patients in clinical cases must employ in their rehabilitation or adaptation to daily life. Finally, when they opened the chest, a note explained what happened with her assistant. Also, within was the last password to enter into the computer program, which freed the students from all the doctor’s assistants, and it stopped the countdown.

To determine which group won the game, the following aspects were considered: 1) If they had managed to escape from the room; 2) If the students had used wildcards or not; and 3) The scores obtained in the two evaluable tests (crossword and clinical case).

### Design and development of surveys

#### Previous survey

Before playing, students were given an anonymous pre-survey on paper to obtain their sociodemographic data such as gender, previous education, university, and age. They were also ask for their perceptions on four statements according to a five-point Likert-type scale (strongly disagree, disagree, neutral, agree, strongly agree) about: traditional classes, flipped classrooms, problem-based learning and gamification. At the end of the questionnaire, an open-ended question was posed as to what their expectations of the activity were, and students were also asked if they had previously participated in any playful or educational escape room.

#### Feedback survey

After playing, students completed an anonymous feedback survey with seven sections: 1) Sociodemographic data (students were prompted to answer questions about their previous education, university, age, and gender, for descriptive data); 2) Gamification (students were asked to indicate their degree of agreement for seven statements about perceptions of gamification. A five-point Likert-type scale (strongly disagree, disagree, neutral, agree, strongly agree) was used); 3) Knowledge (the five questions of a five-point Likert-type scale in this section were focused on the role the escape room may have played in the acquisition or reinforcement of the students’ knowledge as well as the usefulness they believed the game had for their learning; 4) Curricular skills (nine questions having a five point Likert-type scale regarding skills that the students believed they were able to improve during the development of the escape room game); 5) Immersion, engagement and fun (students were asked to determine, on a five-point Likert-type scale, their ratings of how immersed they were in the game); 6) Learning tools, content and materials (explored using a five-point-Likert-type scale. Participants rated their opinion of them with eight statements, from very poor to very good) and 7) Additional comments.

Finally, the four open-ended questions served to obtain the true opinions of the students, without restricting their responses. In this way, modifications and improvements of the game could be established for the future. They were asked about their expectations, what they liked the most, what they liked least and for suggestions.

### Data analysis

All data was coded and entered into Statistical Package for the Social Sciences (SPSS version 24, Softonic S.L., Barcelona, Spain) in preparation for statistical analysis.

#### Tests

The scores in the two trials were compared, before and after the escape game, using parametric and non-parametric statistics to achieve the specific objective initially set (to identify if there are significant differences between individual and / or group learning after the gamification experience).

#### Previous survey

Responses were summarised as percentages to present sociodemographic information related to university, gender, range of age and previous education. The mode and mean score were calculated, and standard deviation determined the level of agreement with the four statements in the five-point Likert-type scale about different ways of giving a seminar. A score of 1 was assigned to strongly disagree, 2 to disagree, 3 to neutral, 4 to agree and 5 to strongly agree.

#### Feedback survey

Consistent with data analysis processes used in *section I*, responses were summarized as percentages to present demographic information about students. For *sections II*, *III, IV, V* and *VI*, questions with a five-point Likert-type scale were used. To the analysis a score of 1 was assigned to strongly disagree, 2 to disagree, 3 to neutral, 4 to agree and 5 to strongly agree. Calculation of the mode, mean score and standard deviation determined the level of agreement with the statements.

### Participants

The “escape room prototype” was played by second year students (*n* = 25, 33.33%) and the “final version” was played by first- and second-year students (*n* = 50, 66.67%). Thus, seventy-five students from occupational therapy university programs participated.

Students from a wide range of backgrounds participated in the escape game. Most participants were ages 18 to 20 (*n* = 62, 82.7%), and age distribution difference was not statistically significant (Chi square of Pearson, *p* = 0.950 > 0.05). There was a predominance of females due to a predominantly female school population (*n* = 58, 77.3%), but the difference in gender distribution was not statistically significant between the three escape rooms (Chi square of Pearson, *p* = 0.158 > 0.05). Education prior to the degree was high school (*n* = 64, 85.3%), vocational training (*n* = 8, 10.7%) or a degree in higher education (*n* = 3, 4%). Again, no statistically significant differences were found (Chi square of Pearson, *p* = 0.684 > 0.05).

### Surveys’ results

#### Pre-game survey

Most students indicated they had never participated in any type of escape game (*n* = 56, 74.7%); only a few of them indicated their participation in other escape rooms (once: *n* = 10, 13.3%; twice: *n* = 2, 2.7%; several: *n* = 7, 9.3%).

**Fig. 4:**
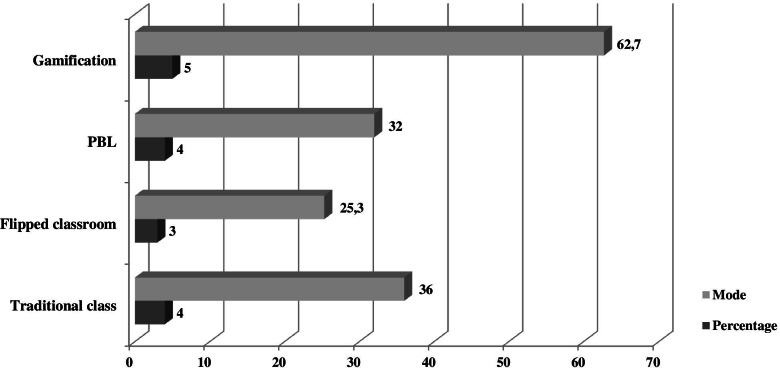
Mode and percentage for each item from pre-game survey

The mean scores of the items referring to the teaching method were compared using a Kruskal-Wallis test for the three escape rooms played. Significant differences were found in the different teaching modalities: traditional class (3.72 ± 1.192; *p* = 0.040 < 0.05), flipped classroom (3.16 ± 1.305; *p* = 0.011 < 0.05) and problem-based learning (3.72 ± 1.134; *p* = 0.002 < 0.05). Although no significant differences were found in gamification (*p* = 0.505 > 0.05), there was a slight preference for it (4.52 ± 0.742) as shown in Fig. 4.

A free-response question asked which kind of games respondents preferred for educational purposes. The students stated quiz games as the favorite (32%), followed by videogames (12%), “any kind of game” and board games (both with 10.7%), interactive games (9.3%) and role playing (8%). Thirteen students did not answer (17.3%). Escape game was a stated favorite among those who preferred interactive games.

Through an open-ended question regarding expectations and previous opinions, four themes were identified: learning, knowledge, motivation, and fun. Frequently repeated expectations were “learning in a fun way”, “testing knowledge” and “teamwork”. Some fragments of the responses that are representative of all the answers are shown (See Table 3):

#### Feedback survey

Only one participant did not answer the feedback survey. As is shown in Table 2, in the first section (gamification assessment) results indicate that students are interested in gamification since assessment of positive wording items ranges from 4.22 (item 6) to 4.41 (item 1), and the mode was 5 (which shows high level of agreement); while the assessment of the 3 negative wording items is 2.78 (item 3), 2.47 (item 4) and 2.79 (item 5), with a mode value of 1 (strongly disagree).

**Table 2:** Results of feedback survey including showing mean (M), standard deviation (SD), and mode (Mo) with frequency (N) and percentage (%)

Gamification: State your level of agreement with the following statements (1 Strongly disagree – 5 Strongly agree)	M (***SD***)	Mo	N (%)
1) I like playing	4.41 (0.905)	5	44 (59.5)
2) I learn while playing	4.23 (0.930)	5	34 (45.9)
3) Educational games are not appropriate for the University	2.78 (1.555)	1	23 (31)
4) Games are a waste of time in class	2.47 (1.546)	1	29 (39.2)
5) I prefer multiple choice test over any educational games	2.59 (1.578)	1	29 (39.2)
6) Games motivate me to learn	4.22 (0.955)	5	33 (44.6)
7) I prefer interactive and team-games	4.30 (0.932)	5	38 (51.4)
**Knowledge:** State your level of agreement with the following statements (1 Strongly disagree – 5 Strongly agree)	**M (*SD*)**	**Mo**	**N (%)**
8) The escape room allowed me to improve my previous knowledge of the subjects	3.93 (0.97)	4	35 (47.3)
9) I was able to apply my knowledge in “anatomy” and “autonomy and functional independence in adults”	3.89 (1.001)	4	34 (45.9)
10) The escape room motivated me to integrate knowledge acquired in other subjects	4.01 (1.027)	4	33 (44.6)
11) Playing the escape room has made me want to learn more about the subjects	3.65 (1.013)	4	29 (39.2)
12) The escape room has been useful for my learning	4.12 (1.006)	4	32 (43.2)
**Curricular skills:** What skills do you think you developed during the game? (1 Strongly disagree – 5 Strongly agree)	**M (*SD*)**	**Mo**	**N (%)**
13) Interpersonal skills (communication)	4.08 (0.888)	4	36 (48.6)
14) Teamwork	4.28 (0.889)	5	36 (48.6)
15) Clinical reasoning	3.96 (0.867)	4	36 (48.6)
16) Problem solving	4.19 (0.917)	4	32 (43.2)
17) Decision making	4.19 (0.902)	4	34 (45.9)
18) Ability to adapt to new situations	4.05 (0.858)	4	37 (50)
19) Planning and time management	3.89 (1.015)	4	32 (43.2)
20) Capacity for analysis and synthesis	3.96 (0.883)	4	36 (48.6)
21) Autonomous learning	3.84 (0.907)	4	32 (43.2)
**Immersion, engagement, and fun:** State level of agreement with the following statements (1 Strongly disagree – 5 Strongly agree)	**M (*SD*)**	**Mo**	**N (%)**
22) While playing I wanted to complete the game	4.62 (0.590)	5	50 (67.6)
23) I wanted to explore all options of the game even if they were false leads	4.36 (0.769)	5	37 (50)
24) Time passed quickly while playing	4.55 (0.705)	5	49 (66.2)
25) I was excited while playing	4.46 (0.762)	5	45 (60.8)
26) I felt like part of the game’s story, being absorbed in it	4.00 (0.936)	5	27 (36.5)
27) The escape room was fun for me	4.38 (0.806)	5	40 (54.1)
**Learning tools, contents, and material:** What is your general option of the tools and contents? (1 Very poor – 5 Very good)	**M (*SD*)**	**Mo**	**N (%)**
28) Therapystein© Software	4.19 (0.902)	5	24 (55.8)
29) Augmented reality + Post-its / QR App	4.15 (1.029)	5	24 (55.8)
30) Puzzles and riddles	4.23 (0.884)	5	20 (46.5)
31) Crosswords	4.14 (0.956)	5	21 (48.8)
32) Clinical cases	4.16 (0.966)	5	19 (44.2)
33) Materials: padlocks, envelops, wildcard, etc.	4.30 (0.840)	5	26 (60.5)
34) Characters and story	4.26 (0.908)	5	20 (46.5)
35) Pictures	4.41 (0.826)	5	25 (58.1)

**Table 3:** Some fragments of the responses that are representative of all the answers

Themes	Examples
Learning	“*Learn the subject*”, “*learn through the game*”, “*learn new knowledge about occupational therapy*”, “*get new knowledge and different methods of approaching different issues and ways of solving problems*” and “*learn teamwork*”.
Knowledge	“*Test our knowledge about the subject*”, “*better understand concepts of the subject and retain better in memory*” and “*my knowledge today is very little, and I hope to strengthen it with the game*”.
Motivation and fun	“*I find it very interesting and important to encourage other kinds of more interactive classes*”, “*I want to learn in a dynamic and fun way*”, “*more enjoyable learning of the subject*”, “*being an activity with a methodology of playfulness is much more striking*”, “*doing an activity of this type requires you participate and get involved in the activity*”, “*we will remember better because we are going to carry out recreational activities*”, “*learn other dynamics when attending seminars*” and “*acquire knowledge without being in front of a book*”.

**Table 4:** Global opinion of the escape room divided by sections

	Assessment				
	Very unfavourably	Unfavourably	Indifferent	Favourably	Very favourably
Knowledge section.	2.7% (n = 2)	4.1% (n = 3)	4.1% (n = 3)	51.4% (*n* = 38)	37.8% (*n* = 28)
Curricular skills section.	–	2.7% (n = 2)	4.1% (*n* = 3)	48.6% (*n* = 36)	44.6% (*n* = 33)
Immersion, engagement, and fun section.	–	–	2.7% (*n* = 2)	27% (n = 20)	70.3% (n = 52)
Learning tools, content, and materials section.	1.4% (n = 1)	–	6.8% (*n* = 5)	21.6% (*n* = 16)	70.3% (*n* = 52)

Items included in the knowledge assessment obtained average scores of 3.65 (item 11) to 4.12 (item 12). The mode was 4 (high level of agreement).

Skills assessment averages ranged 3.84 (item 21) to 4.28 (item 14). The mode of every item was 4, except item 14 (teamwork), which was 5. This suggests that the aim of the escape room was achieved.

Immersion, engagement, and fun averages ranged from 4.00 (item 26) to 4.62 (item 22). The mode of every item was 5 (very high satisfaction).

Learning tools, contents and material obtained averages ranging from 4.14 (item 36) to 4.41 (item 31). The mode of each item was 5, which indicates a high valuation of these. More than half of the students who responded to the survey (54%, *n* = 41) gave a very favourable assessment of gamification as a learning method, and for 27% (*n* = 20) the assessment was favourable (if the three items formulated using negative wording were recoded for the calculation).

Regarding the general opinion of the escape room, 67.6% (*n* = 50) assessed it very favorably, 25.7% (*n* = 19) assessed it favorably and 6.8% (n = 5) assessed it with indifference. In Table 4, the general assessment of each section is shown.

Items were summed to build a composite score with different scale for sections: II (gamification), III (knowledge), IV (curricular skills), V (immersion, engagement, and fun) and VI (learning tools, content, and material). Interestingly, there is a moderate correlation between: sections IV and V (Spearman’s ρ = 0.626, *p* = 0.000 < 0.01), sections III and V (Spearman’s ρ = 0.510, *p* = 0.000 < 0.01), and sections III and IV (Spearman’s ρ = 0.620, *p* = 0.000 < 0.01). Thus, when students are engaged, immersed, and having fun while learning, they devote more time to develop their skills and to apply their knowledge.

After playing the escape room, most students stated having had their expectations exceeded. Stated as positive aspects of the escape: to play as a team, design of the escape room and being able to demonstrate knowledge in the subjects, as well as learning in a fun way.

Data obtained from the open-ended question, “What did you like most about the escape?” can be grouped into the following topics:

Teamwork: Up to twenty-three students recognized that what they liked most was being able to work as a team, collected in the pre-surveys. The following answer is remarkable because it reflects a main aim of the game: “*What I liked the most has been playing on different teams competing to reach something common*”.

Characters, story, and immersion: Students liked how the storylines of the characters became intertwined, capturing their interests till the end. Additionally, they felt like part of the story.

Development, puzzles, riddles, and clues: The search for clues, false clues, the development of the escape, puzzles and riddles, materials and tools have been another of the strengths of the escape room, according to the answers obtained by our students.

Knowledge testing: Several students claimed the escape room served to demonstrate their knowledge as to what they liked the most.

Other: Students liked the development and organization of the activity.

Data obtained from the question “What did you like least about the escape?” can be grouped into the following themes:

Wi-Fi connection: Mentioned two times in the prototype, because it affected the operation of the software program, but was not a problem in the other escape rooms.

Aspects related to the playing of the escape room were mentioned a few times. Because most of the participants had never played an escape game, they did not know how to play. Some students did not read instructions given.

Free AR application or Therapystein QR reader: Although most participants liked the application, some students said they had problems with the application and could not use their phones to obtain clues.

Difficulty level (crossword and clinical cases) was mentioned several times. Some students found theoretical content too difficult because most of them did not study before playing.

### Escape game results and practical application to improve learning

Almost all groups were able to finish and only one of them failed. The mean score of the groups obtained in the escape room was satisfactory (from 3.33 to 9.3). An analysis was performed to estimate the differences between the times the escape room was played based on the average scores of the items using the Kruskal-Wallis-test; there was no significant difference between them (*p* = 0.549 > 0.05).

First, as an indicator of the impact of the educational escape room on the students’ learning, the grades of the second-year students (who attended the escape room prototype; M = 4.67; SD = 1.35) were compared with those students who took the course the preceding year (and did not have the opportunity to participate in the escape game; M = 4.64; SD = 2.08). Although there was an increase in the percentage of marks by 7 points (from 5 to 21.7% of the students presented at exam), no statistically significant differences were found (t-student test, *t* = 0.59, *p* = 0.953 > 0.05), between the exam passing rate (Fisher’s exact test = 1.596, *p* = 0.494 > 0.05) with respect to the students who took the course the preceding year and did not play the escape room prototype.

It should be considered that there were other factors that could have influenced the students’ performance, so it is not realistic to state that the little improvement of students’ performance was due to the escape room. Therefore, the grades of the students who played were not used as an indicator of the impact of the educational escape room on students’ learning. That was the reason why a before and after play test was used to evaluate the content that will be worked on during the game.

After analyzing the data obtained from the test before and after the game, significant differences were detected in the theoretical-practical knowledge after the game. In Anatomy (first year students), a significant improvement range of 46.35% was observed (t-student = − 6.913, *p* = 0.000 < 0.005) if we compare the individual knowledge before and after playing; while in Autonomy and functional independence in the adult (second year students) there was a 22.62% increase in knowledge (t-student = − 5.227, *p* < 0.005).

The learning contents dealt with in the different game tests were, for the most part, general anatomy and osteoarticular pathology terminology. These concepts were asked in the questionnaires, and in the pre-test, they were not known, or a correct answer was not given. After the game, the students answered correctly more frequently, having been able to relate the term asked with the clinical cases and puzzles raised during the game in order to escape. For example, a question posed in the questionnaires consisted of answering: “What is the abduction starter muscle of the arm”, while in the game it was integrated in the case of a patient of Dr. Therapystein who presented rotator cuff syndrome. In this way, the students were able to relate the concepts inside and outside the game.

Because it was a team game, it was considered important to be able to demonstrate the effect of the group, in order to establish if there is a joint enrichment. In Anatomy, a significant improvement range of 25.4% was observed (Wilcoxon test, *p* < 0.005) if we compare the individual and group scores on the knowledge assessment after playing; while in Autonomy and functional independence in the adult (second year students), a significant improvement range of 19.3% was observed (Wilcoxon test, *p* = 0.000 < 0.005). First-year students have more recent anatomical concepts than second-year students. The differences in both individual and group results are because the transversal theoretical contents treated in the escape room were fundamentally based on clinical concepts of an anatomical nature.

## Discussion

Gamification is being widely used to promote learning in classes. Therapystein Visor© App pursues the need for learning through experience and social interaction with both peers and the environment [26]. As well as acquiring a greater knowledge using a fun methodology, they improve curricular skills [10]. Second-year students found the use of the game helpful to remember forgotten terms necessary for patient assessment. On the other hand, first-year students said that the escape room was useful for reviewing all the anatomical terms before the exam. The students of both years had the same opinion that they could apply their knowledge to understand and to solve clinical cases.

With this escape room, students are more goal-oriented by increasing their persistence, learning by repetition, participating in collaboration, and evoking fun and friendly competition with their peers; issues already addressed in previous studies [27, 28] as well as the educational commitment [29]. And although we find in scientific literature escape rooms made in different health disciplines [20, 30, 31], there is no previous literature that uses this type of gamification in the discipline of Occupational Therapy. This may be because it is a relatively new didactic concept that is gaining increasing interest among academics and researchers [32].

Participants showed a high degree of motivation with the escape room, a result found in previous studies [33, 34]. Motivation is an important predictor to improve the academic performance of students and influences the effort and time they spend in the study [35–38]. And even more, motivation is among one of the five fundamental general competencies for the different professional profiles of occupational therapists, together with the ability to work in a multidisciplinary team, possess basic knowledge of the profession, ability to apply knowledge to practice and capacity for analysis and synthesis [39]. This is important to improve the knowledge of occupational therapy students and improve their clinical reasoning regarding anatomical knowledge and its clinical application [6, 23]. According to Rutledge [2], the success of gamification has a positive impact on student motivation, which leads to an increased in study times, improving retention and application [40].

In addition, high scores were obtained in the area of learning tools, contents and material used in the escape room. The fact that the game was in a team and in a collaborative way also increased these scores as in other studies [41–43], as well as improving knowledge in a fun and committed way.

Although, at first, the preparation of the educational escape room requires a great investment of time (even more is needed if not all students can play simultaneously, because everything has to be prepared between sessions), with practice it is easy to adapt new dynamics and mysteries to create new plots and stories that make the experience of the participating students more real. Creating an escape room is challenging and is never complete as there are always ways to improve the game.

Few studies have been done on escape rooms which allow numerous groups of students to play simultaneously [22]. This educational escape room was designed to be of the same duration as regular class lectures or practical learning. Its design allows it to be done in one session using internet connectivity, which significantly reduces the time invested in the event which in turn eases their incorporation into large enrolment courses.

### Study limitations

The Therapystein Visor© App for reading QR codes to search for clues has only been developed and tested on Android mobiles. One of the requirements was that there be one Android mobile per group, a fact that made the teams with more than one Android mobile phone have an advantage, often leading to them being faster at looking for clues.

Another limitation of the study was the small sample size and the composition of the sample of primarily female students, and students between 18 and 20 years old, so study results cannot be extrapolated to the general population. In addition, motivation should have been evaluated and a longitudinal and case-controlled study should have been carried out to see the effectiveness of this type of innovative methodology. The escape room needs more testing and with different genders and age groups to explore its effectiveness further. Therefore, as future lines of research, it is necessary to carry out procedural and strategy studies using gamification as a methodology to improve professional reasoning and their competencies for their practice as future clinicians.

## Conclusions

An increase of improvement in the scores of the theoretical-practical tests is observed which is even more marked after the collaboration as a group when students work together to find a solution for the problems posed. Findings indicated a range of positive experiences and outcomes, and it supports the idea that similar initiatives could be relevant for other courses. Students found the escape room activity to be an effective and innovative learning experience for application of skills, teamwork, and problem solving in a high-pressure situation. Based on these results, it can be suggested that educational escape rooms could have significant positive impacts on student engagement and learning in occupational therapy courses.

On the other hand, it would be interesting to design multidisciplinary escape rooms, with students and professionals from different areas of health sciences, to emulate real situations of their future work in the game. Thus, through play, they could experience real problems under safe conditions; furthermore, PhD students can also benefit from this given similar learning needs.

## Data Availability

The datasets generated and/or analysed during the current study are not publicly available [their information could compromise the privacy of research participants] but are available from the corresponding author on reasonable request.
